# *N* ^4^,*N* ^6^-Dimethyl-5-nitro-*N* ^4^,*N* ^6^-diphenyl­pyrimidine-4,6-diamine

**DOI:** 10.1107/S1600536811035094

**Published:** 2011-09-03

**Authors:** Fuqiang Shi, Li-Hong Zhu, Long Zhang, Ya-Feng Li

**Affiliations:** a School of Chemical Engineering, Changchun University of Technology, Changchun 130012, People’s Republic of China

## Abstract

In the title compound, C_18_H_17_N_5_O_2_, the pyrimidine ring makes dihedral angles of 66.09 (12), 71.39 (13) and 56.7 (3)° with two phenyl rings and the nitro group, respectively. The dihedral angle between the two phenyl rings is 44.05 (14)°.

## Related literature

For applications of pyrimidine diamines, see: Barillari *et al.* (2001 ▶); Che *et al.* (2008 ▶); Itoh *et al.* (2004 ▶); Koppel & Robins (1958 ▶); Shi *et al.* (2011 ▶).
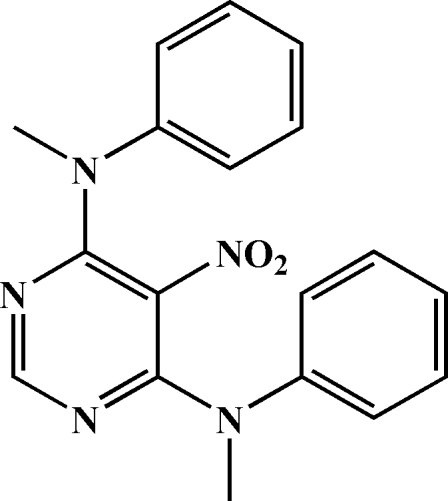


## Experimental

### 

#### Crystal data

C_18_H_17_N_5_O_2_
*M* *_r_* = 335.37
Monoclinic, 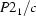

*a* = 10.794 (2) Å
*b* = 7.0019 (14) Å
*c* = 23.650 (6) Å
β = 109.02 (3)°
*V* = 1689.8 (6) Å^3^
*Z* = 4
Mo *K*α radiation
μ = 0.09 mm^−1^
*T* = 293 K
0.50 × 0.12 × 0.10 mm

#### Data collection

Rigaku R-AXIS RAPID diffractometer
Absorption correction: multi-scan (*ABSCOR*; Higashi, 1995 ▶) *T*_min_ = 0.956, *T*_max_ = 0.991
15784 measured reflections
3843 independent reflections
2018 reflections with *I* > 2σ(*I*)
*R*_int_ = 0.108

#### Refinement

*R*[*F* ^2^ > 2σ(*F* ^2^)] = 0.074
*wR*(*F* ^2^) = 0.146
*S* = 1.05
3843 reflections
228 parameters
H-atom parameters constrained
Δρ_max_ = 0.17 e Å^−3^
Δρ_min_ = −0.20 e Å^−3^

### 

Data collection: *PROCESS-AUTO* (Rigaku, 1998 ▶); cell refinement: *PROCESS-AUTO*; data reduction: *CrystalStructure* (Rigaku/MSC, 2002 ▶); program(s) used to solve structure: *SHELXS97* (Sheldrick, 2008 ▶); program(s) used to refine structure: *SHELXL97* (Sheldrick, 2008 ▶); molecular graphics: *DIAMOND* (Brandenburg, 2000 ▶); software used to prepare material for publication: *SHELXL97*.

## Supplementary Material

Crystal structure: contains datablock(s) I, global. DOI: 10.1107/S1600536811035094/is2768sup1.cif

Structure factors: contains datablock(s) I. DOI: 10.1107/S1600536811035094/is2768Isup2.hkl

Supplementary material file. DOI: 10.1107/S1600536811035094/is2768Isup3.cml

Additional supplementary materials:  crystallographic information; 3D view; checkCIF report

## Figures and Tables

**Fig. 1.**
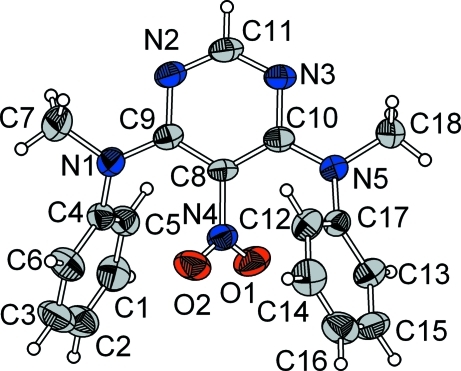
The molecular structure of the title compound, with the atom-labelling scheme. Displacement ellipsoid are shown at the 50% probability level.

## supplementary crystallographic information

### Comment

Pyrimidine diamines, important intermediate products (Koppel *et al.*, 1958; Itoh *et al.*, 2004; Che *et al.*, 2008; Shi *et al.*, 2011), exhibit a wide range of biological activities (Barillari *et al.*, 2001). Here, the crystal structure of *N*^4^,*N*^6^- dimethyl-5-nitro-*N*^4^, *N*^6^-diphenylpyrimidine-4,6-diamine is determined by X-ray single crystal diffraction.

In the structure of the title compound (Fig. 1), the dihedral angles between pyrimidyl and two phenyl rings and between two phenyl rings are 66.09 (12), 71.39 (13) and 44.05 (14)°, respectively.

### Experimental

4,6-Dichloro-5-nitro-pyrimidine (192 mg, 1 mmol), *N*-methylbenzenamine (0.33 mL, 3 mmol) and triethylamine (0.22 mL, 2 mmol) were dissolved in anhydrous THF (10 mL). The reaction mixture was stirred in reflux overnight. The product was concentrated in vacuo, diluted with water, and extracted with EtOAc. The organic phase was washed with 1mol/L HCl, brine, and dried over anhydrous MgSO_4_. The crude product was purified by flash chromatography (elution with 15% EtOAc in petroleum ether) to give *N*^4^,*N*^6^-dimethyl-5-nitro-*N*^4^, *N*^6^-diphenylpyrimidine- 4,6-diamine (yellow solid, 256 mg, 76.4%, 166.4-168.6 °C). ^1^H NMR (CDCl_3_, 400 Hz), δ: 8.46 (s, 1H), 7.23-7.19(m, 4H), 7.13-7.11(m, 2H), 7.02-6.99(m, 4H), 3.50 (s, 6H); ^13^C NMR (CDCl_3_, 100 Hz), δ: 156.0, 155.7, 144.2, 129.2, 126.6, 125.1, 121.0, 42.0; ES-MS: 336.1 [(M + H^+^)].

### Refinement

All H atoms were located from difference Fourier maps. H atoms attached to C atoms were treated as riding [C—H = 0.93–0.96 Å and *U*_iso_(H) = 1.2*U*_eq_(C)].

### Crystal data

**Table:**

C_18_H_17_N_5_O_2_	*F*(000) = 704
*M**_r_* = 335.37	*D*_x_ = 1.318 Mg m^−^^3^
Monoclinic, *P*2_1_/*c*	Mo *K*α radiation, λ = 0.71073 Å
Hall symbol: -P 2ybc	Cell parameters from 500 reflections
*a* = 10.794 (2) Å	θ = 3.1–27.5°
*b* = 7.0019 (14) Å	µ = 0.09 mm^−^^1^
*c* = 23.650 (6) Å	*T* = 293 K
β = 109.02 (3)°	Block, colorless
*V* = 1689.8 (6) Å^3^	0.50 × 0.12 × 0.10 mm
*Z* = 4	

### Data collection

**Table:**

Rigaku R-AXIS RAPID diffractometer	3843 independent reflections
Radiation source: fine-focus sealed tube	2018 reflections with *I* > 2σ(*I*)
graphite	*R*_int_ = 0.108
Detector resolution: 10.00 pixels mm^-1^	θ_max_ = 27.5°, θ_min_ = 3.1°
ω scans	*h* = −14→13
Absorption correction: multi-scan (*ABSCOR*; Higashi, 1995)	*k* = −9→9
*T*_min_ = 0.956, *T*_max_ = 0.991	*l* = −30→30
15784 measured reflections	

### Refinement

**Table:**

Refinement on *F*^2^	Primary atom site location: structure-invariant direct methods
Least-squares matrix: full	Secondary atom site location: difference Fourier map
*R*[*F*^2^ > 2σ(*F*^2^)] = 0.074	Hydrogen site location: inferred from neighbouring sites
*wR*(*F*^2^) = 0.146	H-atom parameters constrained
*S* = 1.05	*w* = 1/[σ^2^(*F*_o_^2^) + (0.0524*P*)^2^ + 0.0764*P*] where *P* = (*F*_o_^2^ + 2*F*_c_^2^)/3
3843 reflections	(Δ/σ)_max_ < 0.001
228 parameters	Δρ_max_ = 0.17 e Å^−^^3^
0 restraints	Δρ_min_ = −0.20 e Å^−^^3^

### Special details

**Table:**

Geometry. All e.s.d.'s (except the e.s.d. in the dihedral angle between two l.s. planes) are estimated using the full covariance matrix. The cell e.s.d.'s are taken into account individually in the estimation of e.s.d.'s in distances, angles and torsion angles; correlations between e.s.d.'s in cell parameters are only used when they are defined by crystal symmetry. An approximate (isotropic) treatment of cell e.s.d.'s is used for estimating e.s.d.'s involving l.s. planes.
Refinement. Refinement of *F*^2^ against ALL reflections. The weighted *R*-factor *wR* and goodness of fit *S* are based on *F*^2^, conventional *R*-factors *R* are based on *F*, with *F* set to zero for negative *F*^2^. The threshold expression of *F*^2^ > σ(*F*^2^) is used only for calculating *R*-factors(gt) *etc*. and is not relevant to the choice of reflections for refinement. *R*-factors based on *F*^2^ are statistically about twice as large as those based on *F*, and *R*- factors based on ALL data will be even larger.

### Fractional atomic coordinates and isotropic or equivalent isotropic displacement parameters (Å^2^)

**Table:**

	*x*	*y*	*z*	*U*_iso_*/*U*_eq_	
O1	0.84317 (18)	0.0703 (3)	0.35192 (9)	0.0577 (6)	
O2	0.85674 (17)	0.2808 (3)	0.42079 (8)	0.0586 (6)	
N1	0.5774 (2)	0.2175 (3)	0.41820 (9)	0.0435 (6)	
N2	0.45045 (19)	0.3519 (3)	0.32882 (10)	0.0434 (6)	
N3	0.52690 (19)	0.3954 (3)	0.24557 (9)	0.0412 (5)	
N4	0.7997 (2)	0.2043 (3)	0.37282 (10)	0.0424 (6)	
N5	0.74616 (19)	0.3627 (3)	0.25459 (9)	0.0393 (5)	
C1	0.7275 (3)	−0.2635 (4)	0.44234 (13)	0.0553 (8)	
H1	0.7216	−0.3782	0.4218	0.066*	
C2	0.8135 (3)	−0.2471 (5)	0.49962 (14)	0.0639 (9)	
H2	0.8663	−0.3498	0.5176	0.077*	
C3	0.8210 (3)	−0.0796 (5)	0.53010 (13)	0.0678 (10)	
H3	0.8784	−0.0692	0.5690	0.081*	
C4	0.6587 (2)	0.0578 (4)	0.44551 (10)	0.0387 (6)	
C5	0.6500 (3)	−0.1108 (4)	0.41512 (11)	0.0446 (7)	
H5	0.5921	−0.1224	0.3763	0.054*	
C6	0.7441 (3)	0.0745 (4)	0.50349 (12)	0.0562 (8)	
H6	0.7496	0.1885	0.5243	0.067*	
C7	0.4741 (3)	0.2699 (5)	0.44293 (13)	0.0705 (10)	
H7A	0.4531	0.4028	0.4354	0.085*	
H7B	0.5041	0.2470	0.4853	0.085*	
H7C	0.3975	0.1943	0.4243	0.085*	
C8	0.6703 (2)	0.2779 (3)	0.33805 (10)	0.0338 (6)	
C9	0.5681 (2)	0.2799 (3)	0.36205 (11)	0.0362 (6)	
C10	0.6494 (2)	0.3447 (3)	0.27970 (11)	0.0359 (6)	
C11	0.4380 (2)	0.3965 (4)	0.27283 (13)	0.0456 (7)	
H11	0.3545	0.4341	0.2493	0.055*	
C12	0.8912 (3)	0.5770 (4)	0.32865 (12)	0.0461 (7)	
H12	0.8181	0.6442	0.3303	0.055*	
C13	0.9840 (3)	0.3278 (4)	0.28633 (12)	0.0474 (7)	
H13	0.9742	0.2256	0.2601	0.057*	
C14	1.0143 (3)	0.6310 (4)	0.36414 (13)	0.0573 (8)	
H14	1.0246	0.7332	0.3904	0.069*	
C15	1.1078 (3)	0.3861 (4)	0.32153 (13)	0.0573 (8)	
H15	1.1815	0.3241	0.3184	0.069*	
C16	1.1223 (3)	0.5339 (5)	0.36078 (13)	0.0604 (9)	
H16	1.2056	0.5690	0.3853	0.072*	
C17	0.8759 (2)	0.4228 (3)	0.29054 (10)	0.0365 (6)	
C18	0.7094 (3)	0.3940 (4)	0.19040 (10)	0.0503 (7)	
H18A	0.6509	0.2945	0.1697	0.060*	
H18B	0.7865	0.3927	0.1786	0.060*	
H18C	0.6664	0.5154	0.1805	0.060*	

### Atomic displacement parameters (Å^2^)

**Table:**

	*U*^11^	*U*^22^	*U*^33^	*U*^12^	*U*^13^	*U*^23^
O1	0.0466 (12)	0.0511 (12)	0.0785 (14)	0.0176 (10)	0.0247 (11)	0.0139 (11)
O2	0.0418 (11)	0.0717 (14)	0.0505 (11)	−0.0089 (10)	−0.0010 (9)	0.0050 (11)
N1	0.0423 (12)	0.0468 (13)	0.0451 (12)	0.0077 (11)	0.0194 (10)	0.0064 (11)
N2	0.0315 (12)	0.0430 (13)	0.0557 (14)	0.0047 (10)	0.0143 (10)	0.0090 (11)
N3	0.0313 (12)	0.0410 (12)	0.0469 (12)	0.0030 (10)	0.0066 (10)	0.0064 (10)
N4	0.0323 (12)	0.0434 (14)	0.0507 (14)	0.0013 (11)	0.0126 (11)	0.0124 (12)
N5	0.0314 (11)	0.0470 (13)	0.0389 (12)	0.0007 (10)	0.0108 (10)	0.0015 (10)
C1	0.065 (2)	0.0434 (16)	0.0620 (18)	0.0039 (15)	0.0270 (16)	0.0043 (15)
C2	0.059 (2)	0.064 (2)	0.066 (2)	0.0128 (17)	0.0162 (16)	0.0262 (18)
C3	0.058 (2)	0.084 (2)	0.0471 (17)	−0.0004 (19)	−0.0025 (15)	0.0157 (18)
C4	0.0346 (14)	0.0434 (15)	0.0364 (13)	−0.0024 (12)	0.0091 (11)	0.0021 (12)
C5	0.0439 (15)	0.0456 (17)	0.0420 (14)	−0.0041 (13)	0.0107 (12)	0.0034 (13)
C6	0.0603 (19)	0.0576 (19)	0.0424 (15)	−0.0027 (15)	0.0054 (14)	−0.0024 (14)
C7	0.069 (2)	0.092 (2)	0.0630 (19)	0.0289 (19)	0.0377 (17)	0.0118 (18)
C8	0.0234 (12)	0.0313 (13)	0.0417 (14)	0.0007 (10)	0.0037 (11)	0.0031 (11)
C9	0.0312 (13)	0.0316 (13)	0.0450 (14)	0.0009 (11)	0.0112 (12)	−0.0002 (12)
C10	0.0302 (13)	0.0311 (13)	0.0424 (14)	−0.0017 (11)	0.0063 (12)	−0.0019 (11)
C11	0.0287 (14)	0.0426 (16)	0.0610 (18)	0.0040 (12)	0.0087 (13)	0.0123 (14)
C12	0.0433 (16)	0.0426 (15)	0.0566 (16)	−0.0036 (13)	0.0219 (13)	−0.0039 (14)
C13	0.0416 (15)	0.0460 (16)	0.0568 (16)	0.0059 (13)	0.0188 (13)	−0.0010 (13)
C14	0.059 (2)	0.0513 (17)	0.0600 (18)	−0.0148 (16)	0.0176 (15)	−0.0084 (15)
C15	0.0347 (15)	0.064 (2)	0.073 (2)	0.0080 (15)	0.0174 (15)	0.0161 (17)
C16	0.0452 (18)	0.068 (2)	0.0580 (18)	−0.0170 (17)	0.0033 (15)	0.0101 (17)
C17	0.0321 (13)	0.0390 (14)	0.0393 (13)	−0.0003 (12)	0.0129 (11)	0.0038 (12)
C18	0.0465 (16)	0.0653 (19)	0.0403 (15)	0.0101 (15)	0.0157 (13)	0.0026 (14)

### Geometric parameters (Å, °)

**Table:**

O1—N4	1.223 (3)	C5—H5	0.9300
O2—N4	1.224 (3)	C6—H6	0.9300
N1—C9	1.370 (3)	C7—H7A	0.9600
N1—C4	1.438 (3)	C7—H7B	0.9600
N1—C7	1.464 (3)	C7—H7C	0.9600
N2—C11	1.324 (3)	C8—C9	1.395 (3)
N2—C9	1.355 (3)	C8—C10	1.404 (3)
N3—C11	1.319 (3)	C11—H11	0.9300
N3—C10	1.353 (3)	C12—C14	1.374 (4)
N4—C8	1.464 (3)	C12—C17	1.382 (3)
N5—C10	1.366 (3)	C12—H12	0.9300
N5—C17	1.444 (3)	C13—C17	1.375 (4)
N5—C18	1.455 (3)	C13—C15	1.385 (4)
C1—C2	1.375 (4)	C13—H13	0.9300
C1—C5	1.381 (4)	C14—C16	1.374 (4)
C1—H1	0.9300	C14—H14	0.9300
C2—C3	1.365 (4)	C15—C16	1.365 (4)
C2—H2	0.9300	C15—H15	0.9300
C3—C6	1.382 (4)	C16—H16	0.9300
C3—H3	0.9300	C18—H18A	0.9600
C4—C5	1.369 (3)	C18—H18B	0.9600
C4—C6	1.386 (3)	C18—H18C	0.9600
			
C9—N1—C4	121.6 (2)	C9—C8—N4	120.7 (2)
C9—N1—C7	118.9 (2)	C10—C8—N4	119.2 (2)
C4—N1—C7	116.6 (2)	N2—C9—N1	116.1 (2)
C11—N2—C9	116.0 (2)	N2—C9—C8	118.9 (2)
C11—N3—C10	115.8 (2)	N1—C9—C8	125.0 (2)
O1—N4—O2	124.5 (2)	N3—C10—N5	117.0 (2)
O1—N4—C8	117.7 (2)	N3—C10—C8	119.1 (2)
O2—N4—C8	117.8 (2)	N5—C10—C8	123.9 (2)
C10—N5—C17	120.2 (2)	N3—C11—N2	129.4 (2)
C10—N5—C18	118.7 (2)	N3—C11—H11	115.3
C17—N5—C18	116.9 (2)	N2—C11—H11	115.3
C2—C1—C5	120.4 (3)	C14—C12—C17	120.0 (3)
C2—C1—H1	119.8	C14—C12—H12	120.0
C5—C1—H1	119.8	C17—C12—H12	120.0
C3—C2—C1	119.8 (3)	C17—C13—C15	119.3 (3)
C3—C2—H2	120.1	C17—C13—H13	120.4
C1—C2—H2	120.1	C15—C13—H13	120.4
C2—C3—C6	120.5 (3)	C16—C14—C12	119.9 (3)
C2—C3—H3	119.8	C16—C14—H14	120.0
C6—C3—H3	119.8	C12—C14—H14	120.0
C5—C4—C6	120.1 (2)	C16—C15—C13	120.5 (3)
C5—C4—N1	120.5 (2)	C16—C15—H15	119.8
C6—C4—N1	119.4 (2)	C13—C15—H15	119.8
C4—C5—C1	119.7 (2)	C15—C16—C14	120.2 (3)
C4—C5—H5	120.1	C15—C16—H16	119.9
C1—C5—H5	120.1	C14—C16—H16	119.9
C3—C6—C4	119.5 (3)	C13—C17—C12	120.1 (2)
C3—C6—H6	120.3	C13—C17—N5	120.0 (2)
C4—C6—H6	120.3	C12—C17—N5	119.9 (2)
N1—C7—H7A	109.5	N5—C18—H18A	109.5
N1—C7—H7B	109.5	N5—C18—H18B	109.5
H7A—C7—H7B	109.5	H18A—C18—H18B	109.5
N1—C7—H7C	109.5	N5—C18—H18C	109.5
H7A—C7—H7C	109.5	H18A—C18—H18C	109.5
H7B—C7—H7C	109.5	H18B—C18—H18C	109.5
C9—C8—C10	120.1 (2)		
